# Quantum correlation in a nano-electro-optomechanical system enhanced by an optical parametric amplifier and Coulomb-type interaction

**DOI:** 10.1038/s41598-023-40490-1

**Published:** 2023-08-23

**Authors:** Habtamu Dagnaw Mekonnen, Tesfay Gebremariam Tesfahannes, Tewodros Yirgashewa Darge, Alemayehu Getahun Kumela

**Affiliations:** 1 Department of Applied Physics, Adama Science and Technology University, P.O.Box 1888, Adama, Ethiopia; 2 Department of Physics, Injibara University, P.O.Box 040, Injibara, Ethiopia; 3 Department of Physics, Arba Minch University, P.O.Box 21, Arba Minch, Ethiopia

**Keywords:** Physics, Quantum physics, Quantum information

## Abstract

In this paper, we investigated the quantum correlation of nano-electro-optomechanical system enhanced by an optical parametric amplifier (OPA) and Coulomb-type interaction. In particular, we consider a hybrid system consisting of a cavity and two charged mechanical oscillators with an OPA, where the optical cavity mode is coupled with a charged mechanical oscillator via radiation pressure, and the two charged mechanical oscillators are coupled through a Coulomb interaction. We use logarithmic negativity to quantify quantum entanglement, and quantum discord to measure the quantumness correlation between the two mechanical oscillators. We characterize quantum steering using the steerability between the two mechanical oscillators. Our results show that the presence of OPA and strong Coulomb coupling enhances the quantum correlations between the two mechanical oscillators. In addition, Coulomb interactions are more prominent in quantum correlations. Besides, in the presence of OPA, the maximum amount of quantum entanglement, quantum steering, and quantum discord were achieved between the two mechanical oscillators is greater than in the absence of OPA. Moreover, a proper phase choice of the optical field driving the OPA enhances quantum correlations under suitable conditions. We obtain quantum entanglement confines quantum steering and quantum discord beyond entanglement. Furthermore, quantum entanglement, quantum steering, and quantum discord decrease rapidly with increasing temperature as a result of decoherence. In addition, quantum discord persists at higher temperature values, although the quantum entanglement between the systems also vanishes completely. Our proposed scheme enhances quantum correlation and proves robust against fluctuations in the bath environment. We believe that the present scheme of quantum correlation provides a promising platform for the realization of continuous variable quantum information processing.

## Introduction

Quantum correlation and entanglement are special types of quantum coherence, and present many charming properties to realize quantum information processing^1–3^. Consequentlly, quantum correlations are widely used in numerous tasks of quantum information processing, for instance, in quantum communication^4^, computation^5^ and metrology^6^. Specifically, quantum entanglement is a key ingredient of quantum information processing that characterize the non-classical property of multipartite quantum systems^7^. Accordingly, numerous researchers show that the nonzero entanglement assures the existence of quantum correlations but the zero entanglement does not assure the absence of quantum correlations in a bipartite quantum state. Unlike the entanglement, the quantum steering can be certifying and judge for creation and verification of optomechanical entanglement^8^. Subsequently, both theoretical and experimental researchers have precisely indicated that the observation of quantum steering is an essential resource in several fundamental applications^9–13^. Thus, the quantum steering is used to quantify how much the two entangled bipartite states are steerable. Such a quantifier exhibits the asymmetric property between two entangled observers (Alice and Bob). In this context, Alice can change (i.e. “steer”) the Bob states by exploiting their shared entanglement^14^. Gaussian quantum discord is another quantum correlation quantifier introduced to go beyond entanglement and measure the non-classical correlations between two subsystems of a quantum system. Therefore, such a quantum discord strives at capturing all the quantum correlations in a bipartite state, including but does not necessarily involve quantum entanglement. Furthermore, the quantum discord is applicable to almost all squeezed-thermal states with nonzero Gaussian discord^15^. It is essentially used in quantifying the quantumness in multiparty systems because of its robustness against decoherence in comparison with entanglement^16^.

Several researchers have studied the quantum correlation between optical and mechanical modes in optomechanical systems^17–21^. Amazioug et al.^22,23^ have been investigated the transfer of quantum correlations from Einstein-Podolsky-Rosen (EPR) entangled squeezed light to the movable mirrors and entanglement, EPR steering, and Gaussian geometric discord in a double cavity optomechanical system. Recently, several schemes for macroscopic entanglement in optomechanical systems have been thoroughly investigated^24–28^. For instance, Vitali et al.^26^ examined stationary entanglement between an optical cavity field mode and a macroscopic vibrating mirror that can be generated using radiation pressure. Furthermore, Zhang et al.^29^ proposed a way to generate stationary entanglement between the cavity mode and the mechanical mode via radiation pressure. While, Yang et al.^30^ have proposed a scheme to generate robust tripartite optomechanical entanglement with a single-cavity optomechanical system driven by a single input laser field. To this aim, some interesting phenomena will occur when an optical parametric amplifier is introduced into an optomechanical cavity, such as the generation of entangled and squeezed states of light^31–34^, enhance mechanical cooling^35^, generate strong mechanical squeezing^36^, and enhance the degree of precision of optomechanical position detection^37^. For instance, Huang et al.^38^ have analyzed the ground state cooling of a macroscopic mechanical oscillator for the quantum manipulation of the mirror by degenerate optical parametric amplifier. In addition, Hu et al.^39^ have examined twofold mechanical squeezing in a cavity optomechanical system that involved an OPA driven by a periodically modulated laser field.

In addition, many schemes have been proposed to generate entanglement in nano-electro-optomechanical systems^40–43^. Specifically, Bai et al^40^ propose a scheme to show that the system consisting of two macroscopic oscillators separated in space which are coupled through Coulomb interaction displays the classical-to-quantum transition behavior under the action of optomechanical coupling interaction. Pan et al.^41^ have studied the entanglement phenomena assisted by a distant nano-electro-optomechanical system with two optical parametric amplifiers. In the presence of OPAs, the degree of entanglement between the two cavity fields is much higher than in the absence of OPAs. The optical parametric amplifier plays a very significant role in the interaction of cavity optomechanics^41^, and the possibility to enhance the radiation pressure at the sum sideband in an optomechanical system containing an OPA has been developed to perform quantum applications^44^. Most recently, Pan et al.^45^ investigated the entanglement phenomena, assisted through an electro-optical hybrid system with an optical parametric amplifier and a Coulomb force interaction, they suggest that the two charged oscillators enhanced the entanglement and output squeezing in an electro-optical hybrid system. Therefore, the presence of OPA, shows how to quantify the quantum correlations in nano-electro-optomechanical systems is still the subject of the active research field. Consequently, searching for smart measurements of the quantum features under a charged mechanical oscillator with an OPA and how to transfer information between the subsystems are active research fields and relatively few studies have been addressed.

In this paper, we investigated quantum correlations such as quantum entanglement, quantum steering, and quantum discord between two charged mechanical oscillators. From the theoretical point of view, our work aim is to contribute to the enhancement of the quantum correlation in the presence of OPA and with strong Coulomb coupling, under three-quantum correlation quantifiers. This shows that our model is different from other previous optomechanical systems that consist of a single charged optomechanical system^28^. Specifically, the hybrid system consists of a cavity and two charged mechanical oscillators with an OPA, where the cavity mode is coupled with a charged mechanical oscillator via radiation pressure, and the two charged mechanical oscillators are coupled through a Coulomb interaction. We thoroughly examine how the nonlinear gain of OPA, the Coulomb coupling strength, the phase of the optical field driving the OPA, and the environmental temperature affect quantum correlations. Our results indicate that the presence of OPA and strong Coulomb coupling enhances the quantum correlations between the two mechanical oscillators. Besides, in the presence of OPA, the maximum amount of quantum entanglement, Gaussian quantum steering, and Gaussian quantum discord achieved between the two mechanical oscillators is greater than in the absence of OPA. This is due to the fact that increasing the nonlinear gain of the OPA increases the photon number in the cavity, which leads to a stronger radiation pressure acting on the left mechanical oscillator. Additionally, we show that a proper phase choice of the optical field driving the OPA enhances quantum correlations under suitable conditions. This is because the proper choice of a phase of the optical field driving the OPA may lead to maximum noise suppression, thereby resulting in maximum quantum correlations. Furthermore, the quantum correlations decline rapidly with increasing temperature as a result of decoherence. Our proposed scheme enhances quantum correlation and proves robust against fluctuations in the bath environment. Therefore, we believe that our results provide a realistic route toward the realization of the quantum correlation under the OPA and Coulomb-type interaction and a framework for future experimentally feasible with the advancement of technology.

The paper is structured as follows. In Section “Model and dynamical equations”, The model and dynamical equations of the system are introduced. In Section “Quantification of quantum correlations”, quantum correlations including quantum entanglement, quantum steering, and quantum discord are discussed. The results of the three kinds of quantum correlations are discussed in Section “Results and discussion”. Conclusions are summarized in Section “Conclusions”.

## Model and dynamical equations

**Figure 1:**
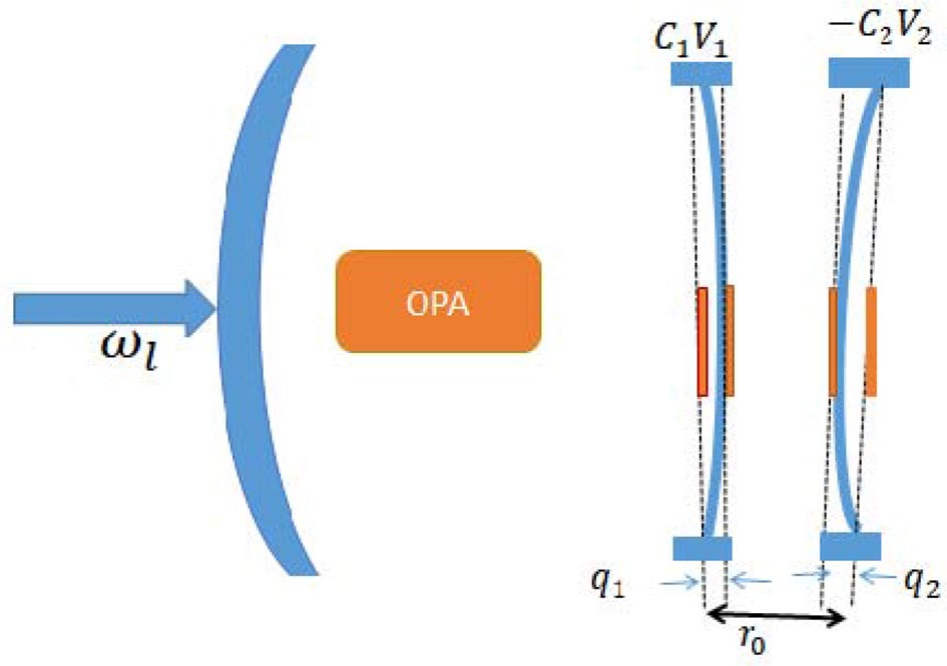
Schematic representation of the system. Hybrid OMS consists of the optical cavity, two charged nanomechanical resonators and a degenerate OPA placed inside the cavity, and the pump of the OPA is not shown.

The hybrid optomechanical system shown in Fig. 1 is composed of a fixed partially transmitting mirror, two charged nano-mechanical oscillators, and an OPA embedded in the cavity. The cavity mode couples the charged nano-mechanical oscillator through the radiation- pressure interaction, and also the first charged nano-mechanical oscillator can be connected to the second spatially separated charged nano-mechanical oscillator by the Coulomb interaction^46^. The cavity is coherently driven by an external laser with frequency $\omega _l$ and amplitude $\Omega$ from the left side of a mirror.  The total Hamiltonian of the system can be written as

1 $$\begin{aligned} {\hat{H}}=\, & {} \hbar \omega _c {\hat{c}}^{\dag }{\hat{c}} +\sum _{i=1}^{2}\frac{\hbar \omega _i}{2}\left( {\hat{p}}^{2}_i + {\hat{q}}^{2}_i\right) -\hbar g q_1 {\hat{c}}^{\dag }{\hat{c}}\nonumber \\{} & {} +i\hbar \Omega \left( {\hat{c}}^{\dag }e^{-i\omega _l t}-{\hat{c}}e^{i\omega _l t}\right) +i\hbar G_a\left( e^{i\theta }{\hat{c}}^{\dag 2} e^{-2i\omega _l t}- e^{-i\theta }{\hat{c}}^{ 2}e^{2i\omega _l t}\right) + {\hat{H}}_{coul}, \end{aligned}$$

where ${\hat{c}}({\hat{c}}^{\dag })$ is the annihilation (creation) operator of the cavity optical mode with cavity frequency $\omega _c$. The first term describes the free energy of the cavity field. The second term denotes the energy of the mechanical modes with frequency $\omega _{i}$, effective mass of mechanical oscillators $m_i$ and the momentum (position) ${\hat{p}}_i ({\hat{q}}_i)$. We define the left mechanical oscillator as mechanical oscillator$_{-}1$ and the right mechanical oscillator as mechanical oscillator$_{-}2$. The third term is the energy of the photon-phonon interaction between the cavity mode and the mechanical oscillator$_{-}1$, with the single photon optomechanical coupling constant $g=\frac{\omega _c}{L} \sqrt{\frac{\hbar }{2m_1\omega _{1}}}$. The fourth term describes the cavity field driven by an input field with frequency $\omega _l$ and amplitude $\Omega =\sqrt{\frac{2\kappa P}{\hbar \omega _l}}$, where $\kappa$ and  *P* are the cavity damping rate and the input laser power, respectively. The fifth term denotes the energy between the OPA and the cavity field, $G_a$ is the nonlinear gain of the OPA, and $\theta$ is the phase of the optical field driving the OPA. The last term shows the Coulomb interaction potential of a charged mechanical oscillators is given as ${\hat{H}}_{coul}=\frac{-C_1V_1C_2V_2}{4\pi \epsilon _0|r_0 +q_1-q_2|}$, where $C_i$ is the gate capacitance, $V_i$ is the voltage of the bias gate, $\epsilon _0$ is the vacuum dielectric constant and $r_0$ is the separation of the equilibrium positions of the two mechanical oscillators.

We assume the distance between the two charged mechanical oscillators is much greater than the small oscillations of the charged mechanical oscillators ($q_1, q_2 \ll r_0$) , the term describing the interaction between two charged mechanical oscillators can be expanded to the second order as^47^

2 $$\begin{aligned} {\hat{H}}_{coul}=-\frac{C_1V_1C_2V_2}{4\pi \epsilon _0r_0}\left( 1-\frac{q_1-q_2}{r_0}+\frac{(q_1-q_2)^2}{r_0^2}\right) , \end{aligned}$$

where the linear terms are absorbed in equilibrium positions, and quadratic terms are incorporated in the renormalization of oscillation frequencies. After omitting the constant term, the Coulomb interaction can be reduced to this form^48,49^

3 $$\begin{aligned} {\hat{H}}_{coul}=\hbar \eta q_1q_2, \end{aligned}$$

where $\eta = \frac{C_1V_1C_2V_2}{4\pi \varepsilon _0 m\omega _{m}r_0^3}$ is Coulomb coupling strength. The Hamiltonian of the system in a rotating frame at the frequency $\omega _l$ takes the form

4 $$\begin{aligned} {\hat{H}}= \hbar \Delta {\hat{c}}^{\dag }{\hat{c}} +\sum _{i=1}^{2}\frac{\hbar \omega _i}{2}\left( {\hat{p}}^{2}_i + {\hat{q}}^{2}_i\right) -\hbar g q_1 {\hat{c}}^{\dag }{\hat{c}} +i\hbar \Omega \left( {\hat{c}}^{\dag }-{\hat{c}}\right) +i\hbar G_a\left( e^{i\theta }{\hat{c}}^{\dag 2} - e^{-i\theta }{\hat{c}}^{ 2}\right) +\hbar \eta q_1q_2, \end{aligned}$$

where $\Delta = \omega _c -\omega _l$ is optical cavity detuning. Using Eq. (4) and considering the damping and noise terms into Heisenberg equations, we can obtain the quantum Langevin equations of the hybrid system as

5 $$\begin{aligned} \dot{{\hat{q}}}_1=\, & {} \omega _1{\hat{p}}_1, \end{aligned}$$

6 $$\begin{aligned} \dot{{\hat{p}}}_1=\, & {} -\omega _1{\hat{q}}_1 + g {\hat{c}}^{\dag }{\hat{c}} - \eta {\hat{q}}_2 -\gamma _1 {\hat{p}}_1 +\xi _1(t), \end{aligned}$$

7 $$\begin{aligned} \dot{{\hat{q}}}_2=\, & {} \omega _2{\hat{p}}_2, \end{aligned}$$

8 $$\begin{aligned} \dot{{\hat{p}}}_2=\, & {} -\omega _2{\hat{q}}_2 - \eta {\hat{q}}_1 -\gamma _2 {\hat{p}}_2 +\xi _2(t), \end{aligned}$$

9 $$\begin{aligned} \dot{{\hat{c}}}=\, & {} -(\kappa + i\Delta -ig{\hat{q}}_1){\hat{c}} +2G_a {\hat{c}}^{\dag }e^{i\theta }+\Omega +\sqrt{2\kappa } {\hat{c}}_{in}, \end{aligned}$$

where $\gamma _1(\gamma _2)$ is the damping rate of the two mechanical oscillators and ${\hat{c}}_{in}$ is the input vacuum noise operator with zero mean value and nonzero correlation function $\langle {\hat{c}}_{in}(t){\hat{c}}^{\dag }_{in}(t')\rangle = \delta (t-t')$^26,27^. The quantum Brownian noise operators $\xi _1(t)$ ($\xi _2(t)$) a rise from the coupling between the two mechanical oscillators with the environment, and their mean values are zero and correlation function

10 $$\begin{aligned} \langle \xi _i(t) \xi _i(t')\rangle = \frac{\gamma _i}{\omega _{i}}\int \frac{d\omega _i}{2\pi } e^{-i\omega _i(t-t')} \omega _{i}\left( 1+coth\left( \frac{\hbar \omega _i}{2\kappa _B T}\right) \right) , i=1,2. \end{aligned}$$

where $\kappa _B$ is the Boltzmann constant and *T* is the temperature of the reservoir related to the mechanical oscillators. However, quantum effects are revealed just for the oscillators with a large quality factor $Q_i= \frac{\omega _i}{\gamma _i}\gg 1$. In this limit, the correlation function of the noise $\xi _i(t)$ can be written as^50^

11 $$\begin{aligned} \langle \xi _i(t)\xi _i(t')+\xi _i(t')\xi _i(t)\rangle /2=\gamma _i(2{\bar{n}}+1)\delta (t-t'). \end{aligned}$$

In which, ${\bar{n}}= (exp(\frac{\hbar \omega _i}{\kappa _B T}-1))^{-1}$ is the mean thermal phonon number. We utilize the nonlinear quantum Langevin equations for the optical mode and the two mechanical oscillators by taking into account the condition of intense laser driving and weak coupling, i.e., $\omega _l \gg \kappa \gg g$^51^. We use the linearization approach, expanding each field operator as the sum of its steady-state mean values and fluctuation operator, which can be treated separately as ${\hat{c}}= c^s +\delta {\hat{c}}$, ${\hat{q}}_i= q_i^s +\delta {\hat{q}}_i$,  ${\hat{q}}_i= q_i^s +\delta {\hat{p}}_i$, where  $c^s$, $q_i^s$ and $p_i^s$ are the mean values for operators   ${\hat{c}}$,  ${\hat{q}}_i$ and ${\hat{p}}_i$. The steady-state mean values of the operators can easily be obtained by setting all time derivatives equal to zero in Eqs. (5–9) such that the steady-state mean value for the hybrid system can be obtained

12 $$\begin{aligned}{} & {} p_{1}^s=p_{2}^s=0, ~~q_{1}^s = \frac{-\omega _2~ g|c^s|^2 }{\eta ^2 - \omega _1\omega _2},~~~q_{2}^s = \frac{ g|c^s|^2 \eta }{\eta ^2 - \omega _1\omega _2}, \end{aligned}$$

13 $$\begin{aligned}{} & {} c^s = \frac{\kappa -i\Delta '+2G_a e^{i\theta }}{\kappa ^2 +\Delta '^2 - 4G_a ^2} \Omega , \end{aligned}$$

where $\Delta '=\Delta -g q^s_{1}$ is the effective cavity detuning from the frequency of the input laser in the presence of the radiation pressure. In order to examine the quantum correlation between the two mechanical oscillators, we need to calculate the fluctuations of their corresponding operators. The cavity is intensively driven with a very large input laser power, which means that at the steady state, the intracavity field has a large amplitude, i.e., $|c^s|\gg 1$. Under, the strong driving limit, here we have neglected the high-order small terms of the fluctuation part, the linearized quantum Langevin equations can be written as

14 $$\begin{aligned} \delta {\dot{{\hat{q}}}}_1=\, & {} \omega _1\delta {{\hat{p}}}_1, \end{aligned}$$

15 $$\begin{aligned} \delta {\dot{{\hat{p}}}}_1=\, & {} -\omega _1\delta {{\hat{q}}}_1 +g (c^{*s}\delta {{\hat{c}}} +c^s\delta {{\hat{c}}^{\dag }}) - \eta \delta {{\hat{q}}}_2-\gamma _1 \delta {{\hat{p}}}_1 +\xi _1(t), \end{aligned}$$

16 $$\begin{aligned} \delta {\dot{{\hat{q}}}}_2=\, & {} \omega _2 \delta {{\hat{p}}}_2, \end{aligned}$$

17 $$\begin{aligned} \delta {\dot{{\hat{p}}}}_2=\, & {} -\omega _2\delta {{\hat{q}}}_2 - \eta \delta {{\hat{q}}}_1 -\gamma _2 \delta {{\hat{p}}}_2 +\xi _2(t), \end{aligned}$$

18 $$\begin{aligned} \delta {\dot{{\hat{c}}}}=\, & {} - (\kappa + i\Delta ')\delta {{\hat{c}}} +igc^s\delta {{\hat{q}}}_1 + 2G_a e^{i\theta } \delta {{\hat{c}}^{\dag }} +\sqrt{2\kappa }\delta {{\hat{c}}_{in}}. \end{aligned}$$

We choose the phase reference of the cavity field $c^s$ is real. In order to study the quantum statistical properties of the system through the small fluctuations of the system around the steady-state regime. Specifically, the full information about the correlations and entanglement can be characterized by analyzing the variance between the quadrature components of the fields. For this purpose, we define dimensionless quadrature operators. We define the quadrature operators for each mode as $\delta {{\hat{x}}} =(\delta {{\hat{c}}^{\dag } + \delta {{\hat{c}}}})/\sqrt{2}$ ,  $\delta {{\hat{y}}} =i(\delta {{\hat{c}}^{\dag } - \delta {{\hat{c}}}})/\sqrt{2}$,  and the corresponding Hermitian input noise operators are  $\delta {{\hat{x}}}_{in} =(\delta {{\hat{c}}^{\dag }}_{in} + \delta {{\hat{c}}}_{in})/\sqrt{2}$,   $\delta {{\hat{y}}}_{in} =i(\delta {{\hat{c}}^{\dag }}_{in} - \delta {{\hat{c}}}_{in})/\sqrt{2}$.  Thus, we obtain the linearized quantum fluctuations equations

19 $$\begin{aligned} \delta {\dot{{\hat{q}}}}_1=\, & {} \omega _1\delta {{\hat{p}}}_1, \end{aligned}$$

20 $$\begin{aligned} \delta {\dot{{\hat{p}}}}_1=\, & {} -\omega _1\delta {{\hat{q}}}_1 - \eta \delta {{\hat{q}}}_2 + G_0\delta {{\hat{x}}} -\gamma _1 \delta {{\hat{p}}}_1 +\xi _1(t), \end{aligned}$$

21 $$\begin{aligned} \delta {\dot{{\hat{q}}}}_2=\, & {} \omega _2\delta {{\hat{p}}}_2, \end{aligned}$$

22 $$\begin{aligned} \delta {\dot{{\hat{p}}}}_2=\, & {} -\omega _2\delta {{\hat{q}}}_2 - \eta \delta {{\hat{q}}}_1 -\gamma _2\delta {{\hat{p}}}_2 +\xi _2(t), \end{aligned}$$

23 $$\begin{aligned} \delta {\dot{{\hat{x}}}}=\, & {} - (\kappa -2G_a cos\theta ) \delta {{\hat{x}}} + (\Delta ' +2G_a sin\theta ) \delta {{\hat{y}}} +\sqrt{2\kappa }\delta {{\hat{x}}_{in}}, \end{aligned}$$

24 $$\begin{aligned} \delta {\dot{{\hat{y}}}}=\, & {} -(\Delta '- 2G_a sin\theta ) \delta {{\hat{x}}}- (\kappa + 2G_a cos\theta ) \delta {{\hat{y}}} +\sqrt{2\kappa }\delta {{\hat{y}}_{in}}, \end{aligned}$$

where $G_0= \sqrt{2}g c^s$ is the effective optomechanical coupling. The equations of motion for the quantum fluctuations from Eqs. (19)–(24) can be written in a compact form

25 $$\begin{aligned} {\dot{u}}(t)= A u(t)+n(t), \end{aligned}$$

where  $u(t)= (\delta {\hat{q}}_{1},\delta {\hat{p}}_{1},\delta {\hat{q}}_{2}, \delta {\hat{p}}_{2},\delta {\hat{x}},\delta {\hat{y}})^T$ and $n(t)=(0,\xi _1(t),0,\xi _2(t),\sqrt{2\kappa }\delta {\hat{x}}_{in}, \sqrt{2\kappa }\delta {\hat{y}}_{in})^T$ are the column vector of the fluctuation and the column vector of the noises sources, respectively. The drift matrix *A* can be defined by

26 $$\begin{aligned} A=\left( \begin{array}{c c c c c c } 0 &{}\quad \omega _1&{}\quad 0 &{}\quad 0&{}\quad 0&{}\quad 0 \\ -\omega _ 1&{}\quad -\gamma _1 &{}\quad -\eta &{}\quad 0 &{}\quad G_0&{}\quad 0 \\ 0&{}\quad 0 &{}\quad 0&{}\quad \omega _ 2&{}\quad 0 &{}\quad 0 \\ -\eta &{}\quad 0 &{}\quad -\omega _ 2 &{}\quad -\gamma _2&{}\quad 0&{}\quad 0\\ 0&{}\quad 0&{}\quad 0&{}\quad 0 &{}\quad -\kappa + 2G_acos\theta &{}\quad \Delta '+ 2G_a sin\theta \\ G_0&{}\quad 0&{}\quad 0&{}\quad 0&{}\quad - \Delta '+ 2G_a sin\theta &{}\quad -\kappa - 2G_acos\theta \end{array}\right) . \end{aligned}$$

Therefore, the drift matrix A of Eq. (26) contains all the information about the system. It is worth noting that the system can achieve a stable steady-state condition when all of the real parts of the eigenvalues of the drift matrix A are negative. The stability condition can be obtained by using the Routh-Hurwitz criterion^52^. Therefore, the steady state of the quantum fluctuations is a continuous variable Gaussian state. This state is fully characterized by a 6$\times$6 covariance matrix (CM) with corresponding components defined as

27 $$\begin{aligned} R_{ij}= \langle u_{i}(t)u_{j}(t')+ u_{j}(t)u_{i}(t')\rangle /2. \end{aligned}$$

Accordingly, we can express the above as

28 $$\begin{aligned} R_{ij}= \sum _{ij}\int _{0}^{\infty } dt\int _{0}^{\infty } dt' f(t)_{ij}f(t')_{ji} \chi (t-t')_{ij}, \end{aligned}$$

where $f(t)=exp(At)$, and as $t\rightarrow \infty$ , the system is stable. $\chi (t-t')_{ij}= \langle n(t)_i n(t')_j + n(t')_j n(t)_i\rangle /2$ is the matrix of the stationary noise correlation functions. As a consequence, and using the fact that the components of *n*(*t*) are uncorrelated, and using Eq. (11), then we get $\chi (t-t')_{ij} =D_{ij}\delta (t-t')$, where $D=diag(0,\gamma _1(2{\bar{n}}+1),0, \gamma _2(2{\bar{n}}+1),\kappa ,\kappa )$ is the noise correlation matrix. The solution of Eq. (28) becomes $R=\int _{0}^{\infty } dt f(t)Df(t)^T$. The stability conditions of the systems are satisfied, then the steady-state correlation matrix can be derived by considering the Lyapunov equation^53^

29 $$\begin{aligned} AR+RA^T=-D. \end{aligned}$$

The correlation matrix *R* can be written in the form of a block matrix

30 $$\begin{aligned} R=\left( \begin{array}{c c c } K_{m_1} &{}\quad L_{m_1m_2}&{}\quad L_{cm_1} \\ {L^{T}_{m_1m_2}}&{}\quad K_{m_2} &{}\quad L_{cm_2} \\ {L^T_{cm_1}}&{}\quad {L^T_{cm_2}} &{}\quad K_{c} \end{array}\right) , \end{aligned}$$

where each block represents a $2\times 2$ matrix. The blocks on the diagonal represent variance within each subsystem (the optical cavity mode, the two mechanical modes ), whereas the blocks off the diagonal represent the correlations between subsystems.

## Quantification of quantum correlations

In this section, we measure the quantum correlation between the subsystems. To this aim, it is commonly accepted that this quantum correlation transfer provides a potential tool to exploit the quantum information encoded in mechanical modes that can be more resilient against decoherence effects. The enhancement of this transfer is crucial. In this sense, we measure the quantum correlation between the subsystems through three quantum quantifiers such as quantum entanglement, quantum steering, and quantum discord. Particularly, the logarithmic negativity $E_N$ is a witness of the entanglement between the bipartite subsystems in a continuous variable system. It is defined by^54,55^

31 $$\begin{aligned} E_N= max[0,-log 2{\nu _s}], \end{aligned}$$

which $\nu _s$ is the smallest partial transposed symplectic eigenvalue $\chi$ and given by

32 $$\begin{aligned} \nu _s= \left[ \frac{\Lambda -\sqrt{\Lambda ^2-4det\chi }}{2}\right] ^{\frac{1}{2}}, \end{aligned}$$

where $\Lambda = det K_{m_1}+ det K_{m_2} -2det L_{m_1m_2}$, and the correlation matrix is associated with the selected bipartite, and by neglecting the rows and columns, we obtained the interesting mode from Eq. (30) in the form of $2\times 2$, block matrix

33 $$\begin{aligned} \chi = \left( \begin{array}{cc} K_{m_1} &{}L_{m_1m_2} \\ L_{m_1m_2}^T&{}K_{m_2} \end{array}\right) . \end{aligned}$$

**Figure 2:**
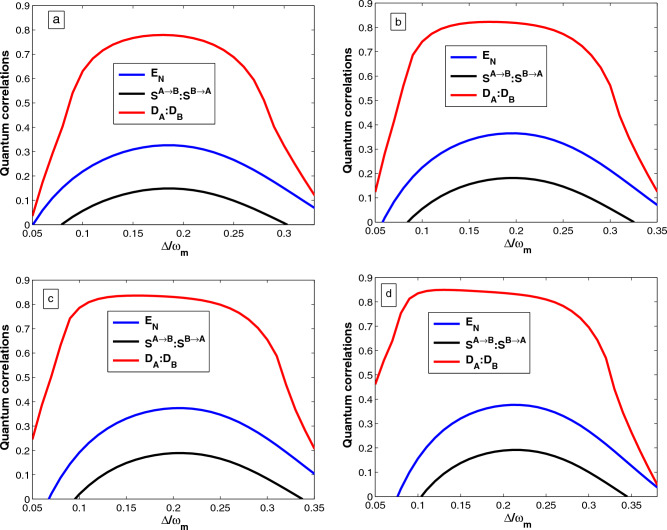
Plots of quantum correlations, e.g., logarithmic negativity $E_N$, quantum steering($S^{A\rightarrow B}:S^{B\rightarrow A}$), and quantum discord($D_{A}:D_{B}$) as function of the normalized detuning $\Delta /\omega _m$ for different value of the nonlinear gain of the OPA (**a**) $G_a=0$, (**b**) $G_a=0.26\kappa$ , (**c**) $G_a=0.35\kappa$, and (**d**) $G_a=0.4\kappa$ with phase of the optical field driving the OPA ($\theta = 0$), temperature ($T =4$ mK), laser power $(P =10~\textrm{mW})$, and Coulomb coupling strength $(\eta = 0.95\omega _m)$. Other parameters are listed in the main text.

**Figure 3:**
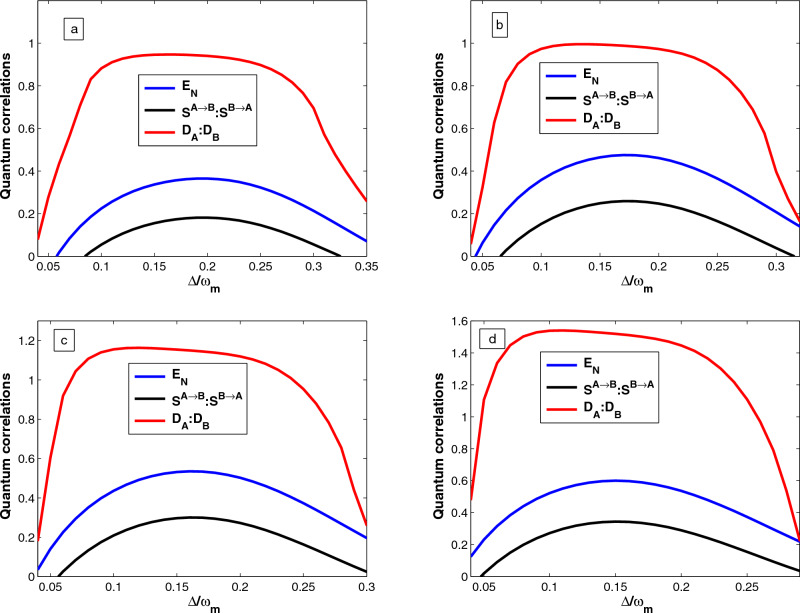
Plots of quantum correlations, e.g., logarithmic negativity $E_N$, quantum steering  ($S^{A\rightarrow B}:S^{B\rightarrow A}$), and quantum discord ($D_{A}:D_{B}$) as a function of the normalized detuning $\Delta /\omega _m$ for different value of Coulomb coupling strength (**a**) $\eta =0.95\omega _m$, (**b**) $\eta =0.97\omega _m$ , (**c**) $\eta =0.98\omega _m$, and (**d**) $\eta =0.99\omega _m$ with the nonlinear gain of the OPA $(G_a = 0.26\kappa )$. Other parameters are the same as Fig. 2.

**Figure 4:**
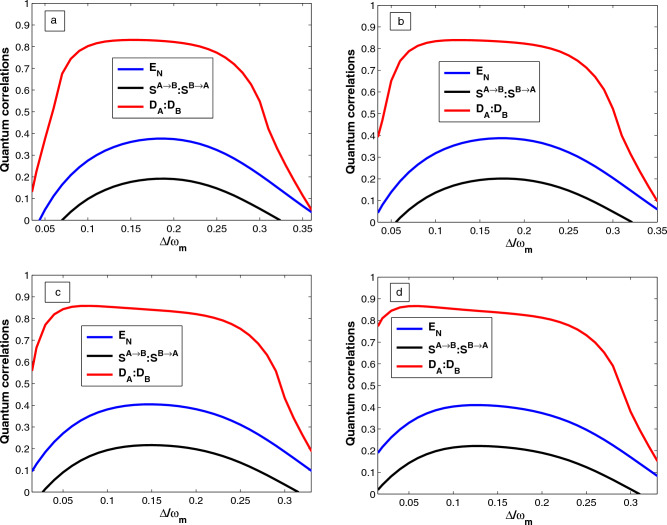
Plots of quantum correlations, e.g., logarithmic negativity $E_N$, quantum steering ($S^{A\rightarrow B}:S^{B\rightarrow A}$), and quantum discord ($D_{A}:D_{B}$) as function of the normalized detuning $\Delta /\omega _m$ for different value of phase of the optical field driving the OPA (**a**) $\theta =\pi /16$, (**b**) $\theta =\pi /8$ , (**c**) $\theta =\pi /4$, and (**d**) $\theta =\pi /3$ with the nonlinear gain of the OPA $(G_a = 0.26\kappa )$. Other parameters are the same as Fig. 2.

**Figure 5:**
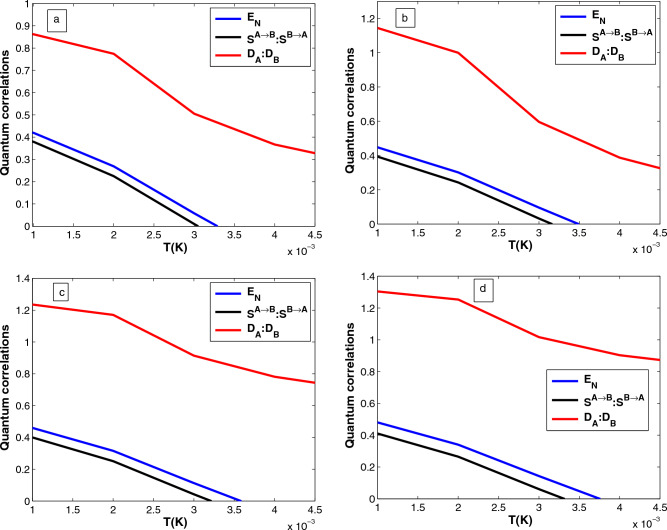
Plots of quantum correlations, e.g., logarithmic negativity $E_N$, quantum steering ($S^{A\rightarrow B}:S^{B\rightarrow A}$), and  quantum discord ($D_{A}:D_{B}$) as a function of the temperature for different values drive laser power (**a**) $P=50\textrm{mW}$, (**b**) $P=70 \textrm{mW}$, (**c**) $P=80~\textrm{mW}$, and (**d**) $P=100~\textrm{mW}$ with $\Delta =0.6\omega _m$ with the nonlinear gain of the OPA $(G_a = 0.26\kappa )$. The remaining set of parameters is the same as Fig. 2.

Furthermore, we can see that the necessary and sufficient condition for the Gaussian state being entangled if $\nu _s < 0.5$, which is entirely identical to Simon’s criterion, which states that the necessary and sufficient condition for entanglement of non positive partial transpose condition for Gaussian states^56^. Consequently, we numerically describe the results of those calculations via plotted in Figs. 2, 3, 4 and 5. Furthermore, quantum steering is another quantum correlation quantifier that is an essential resource in several fundamental applications^12,13^. It is a measure of asymmetric property between two entangled observers (between the mechanical oscillator$_{-}1$ and mechanical oscillator$_{-}2$ in this case). Moreover, it provides a way to quantify how much steerability the two separate mechanical oscillators possess. If we consider the information transfer between the mechanical oscillator$_{-}1$ and mechanical oscillator$_{-}2$ due to their correlation and label Alice (A: mechanical oscillator$_{-}1$ ) and Bob (B: mechanical oscillator$_{-}2$ ), we use the covariance matrix of mechanical oscillators of Eq. (33) and the steerability of mechanical oscillator$_{-}2$ by mechanical oscillator$_{-}1$  $A\rightarrow B$ is defined as ^14^

34 $$\begin{aligned} S^{A\rightarrow B}=max\left[ 0,-ln({\bar{v}}^B)\right] , \end{aligned}$$

where ${\bar{v}}^B$ is the symplectic eigenvalues of $\sqrt{det \chi }$ and $\chi = K_{m_2} -L_{m_1m_2}^TK_{m_1}^{-1}L_{m_1m_2}$, derived from the Schur complement of $K_{m_1}$ in the covariance matrix $\chi$. The measure of Gaussian quantum steering of mechanical oscillator$_{-}2$ by mechanical oscillator$_{-}1$ is given by

35 $$\begin{aligned} S^{A\rightarrow B}=max\left[ 0,\frac{1}{2}ln\left( \frac{det K_{m_1} }{4det \chi }\right) \right] . \end{aligned}$$

The corresponding measure of the Gaussian steerability $B\rightarrow A$ can be found by

36 $$\begin{aligned} S^{B\rightarrow A}=max\left[ 0,\frac{1}{2}ln\left( \frac{det K_{m_2}}{4det \chi }\right) \right] . \end{aligned}$$

From the above, they are two possibilities for quantum steering between A and B: If $S^{A\rightarrow B}=S^{B\rightarrow A}=0$, there is no-way steering, which means Alice cannot steer Bob and vice versa even if they are not separable, and two-way steering if $S^{A\rightarrow B}=S^{B\rightarrow A} > 0$. In actuality, a non-separable state is not always a steerable state, while a steerable state is always not separable.

Finally, we use another quantum correlation quantifier which is a fundamental notion allowing for the description of the quantumness of the correlations present in the state of a quantum system. In our case, the Gaussian quantum discord denotes non-classical correlations if the mechanical oscillator$_{-}1$ and mechanical oscillator$_{-}2$ are separable or not. Employing Eq. (33), the Gaussian quantum discord for the mechanical oscillator$_{-}1$ defined as^57^

37 $$\begin{aligned} D_A= f\left( \sqrt{det K_{m_2}}\right) -f(\Gamma _+)-f(\Gamma _-)+f(\varepsilon ), \end{aligned}$$

The simplectic eigenvalues given by

38 $$\begin{aligned} \Gamma _{\pm }= \left[ \frac{\Lambda ' \pm \sqrt{\Lambda '^2-4det\chi }}{2}\right] ^{\frac{1}{2}}, \end{aligned}$$

with $\Lambda '= det (K_{m_1})+ det (K_{m_2}) +2det (L_{m_1m_2})$ and    $\epsilon$  is defined by

39 $$\begin{aligned} \varepsilon = \frac{\sqrt{det(K_{m_1})}+2\sqrt{det (K_{m_1}) det (K_{m_2})}+2det (L_{m_1m_2})}{1+2\sqrt{det (K_{m_1})}}. \end{aligned}$$

Similarly, the quantum discord for the mechanical oscillator$_{-}2$ can be found as

40 $$\begin{aligned} D_B= f\left( \sqrt{det K_{m_1}}\right) -f(\Gamma _+)-f(\Gamma _-)+f(\varepsilon '), \end{aligned}$$

where

41 $$\begin{aligned} \varepsilon '= \frac{\sqrt{det(K_{m_2})}+2\sqrt{det (K_{m_1}) det (K_{m_2})}+2det (L_{m_1m_2})}{1+2\sqrt{det (K_{m_2})}}, \end{aligned}$$

where the function *f* is defined by

42 $$\begin{aligned} f(x)=\left( x+\frac{1}{2}\right) ln\left( x+\frac{1}{2}\right) -\left( x-\frac{1}{2}\right) ln\left( x-\frac{1}{2}\right) . \end{aligned}$$

The quantum state of the mechanical oscillator$_{-}1$ and mechanical oscillator$_{-}2$ are not separable if   $D_{A}> 1:D_{B} > 1$ . Moreover, if the condition   $0 \le D_{A}< 1: 0 \le D_{B} < 1$  is satisfied, the mechanical oscillator$_{-}1$ and mechanical oscillator$_{-}2$ can be in a separable state or an entangled state. Thus, we numerically describe the results of those calculations and plotted in Figs. 2, 3, 4 and 5.

## Results and discussion

In this section, we investigate the quantum correlation through an optical parametric amplifier and coulomb-type interaction in the hybrid system. This can be understood analytically by inspecting the structure of the drift matrix A of Eq. (26) which contains all the information about the system. Specifically, we have employed a three-quantum correlation quantifier’s expression of Eqs. (31), (35), and (37). Moreover, to obtain the covariance matrix, we numerically solve Eq. (29) and numerically calculate the logarithmic negativity, which is used as the witness of quantum entanglement, and Gaussian quantum discord that gives the measure of all non-classical correlations. Furthermore, we numerically calculate the quantum steering to characterize the steerability between the two mechanical modes. We now investigate the properties of the quantum correlation of the hybrid system. For simplicity, we assume the parameters of the two mechanical oscillators are identical, i.e., $\omega _1 = \omega _2 = \omega _m$, $\gamma _1 = \gamma _2 = \gamma _m$ and  $T_1=T_2= T$. The parameters used in our numerical calculations are chosen based on the experiment conditions reported in ^58,59^ where, $\omega _m = 200\pi$ MHz, $\gamma _m = 20\pi$ Hz, $\kappa = 88.1$ MHz, $m = 5$ ng, $L = 1$ mm, and the wavelength of driving laser $\lambda = 810$ nm.

In Fig. 2, we report the quantum correlations measuring between the two mechanical oscillators using logarithmic negativity $E_N$, quantum steering ($S^{A\rightarrow B}: S^{B\rightarrow A}$), and quantum discord ($D_{A}:D_{B}$) as a function of the normalized detuning $\Delta /\omega _m$ for different values of nonlinear gain of the OPA (a) $G_a=0$, (b) $G_a=0.26\kappa$, (c) $G_a=0.35\kappa$, and (d) $G_a=0.4\kappa$. In Fig. 2a–d, we numerically display the effect of the nonlinear gain of the OPA on the quantum entanglement, quantum steering, and quantum discord between the two mechanical oscillators. We generate the bipartite entanglement, quantum steering, and quantum discord between the two mechanical modes as functions of normalized detuning for different values of the nonlinear gain of the OPA. In particular, using realistic parameters for which a significant amount of quantum correlation is achievable. Specifically, one can observe that a correlation exists between the two oscillators, implying that there is a quantum correlation between them, even though they are separated. Furthermore, Fig. 2a–d, shows one can clearly understand that the higher the nonlinear gain of the OPA coupling parameter is the stronger the oscillators entangle and the broader the range of the correlation between the subsystems can be realized. This shows that increasing the nonlinear gain of the OPA enhances quantum entanglement, quantum steering, and quantum discord compared to the absence of the OPA, i,e., under ordinary light driving. Our result shows that if there is no Coulomb coupling, it is impossible to entangle the two separated oscillators. This is because increasing the nonlinear gain of the OPA corresponds to the increase in the photon number in the optical cavity, which leads to a stronger radiation pressure acting on the mechanical oscillator$_{-}1$ and the Coulomb coupling between the mechanical oscillators. Furthermore, our results are consistent with those reported in^45^.

Next, we explore the crucial role of Coulomb coupling strength $\eta$ on the quantum correlations, e.g., quantum entanglement, quantum steering, and quantum discord between the two mechanical oscillators separated in space. Figure 3, we plot the logarithmic negativity $E_N$, quantum steering ($S^{A\rightarrow B}:S^{B\rightarrow A}$), and quantum discord ($D_{A}:D_{B}$) as function of the normalized detuning $\Delta /\omega _m$ for different value of Coulomb coupling strength (a) $\eta =0.95\omega _m$, (b) $\eta =0.97\omega _m$ , (c) $\eta =0.98\omega _m$, and (d ) $\eta =0.99\omega _m$. As illustrated in the previous section, as long as the logarithmic negativity which characterizes the entanglement remains positive, there is an entanglement between the oscillators, meaning that there is a quantum correlation between two mechanical oscillators, even though they are separated in space. As can be seen from Fig. 3a–d that the presence of strong Coulomb coupling enhances the entanglement, quantum steering, and quantum discord of mechanical oscillators separated by space. It is worthwhile to point out that the larger the coulomb coupling results the more strongly entangled the mechanical oscillators. Furthermore, if there is no Coulomb coupling strength, it is impossible to entangle the two oscillators separately. Therefore, the Coulomb interaction between the two oscillators is the most essential parameter to realize the state transfer between the subsystems. Furthermore, our results are consistent with those reported in^40,45^. Now, we consider the feasibility of the choice of the numerical value of the coupling strength $\eta$ in the experiment. If we apply the reported experimental parameters, i.e., the gate voltage $V_1 = V_2 = 200V$, the capacitance of the gate $C_1 = C_2 = 2.4nF$ and the separation between mechanical oscillators without coulomb and optomechanical interaction $r_0 =160 \upmu \textrm{m}$^40,47^, in this situation $\eta \approx 0.33\omega _m$. If we compare the numerical values used in our coupled optomechanical system, it is obvious that our choice of the numerical value of coulomb coupling strength is easily executable in experiments. Thus, our hybrid optomechanical system can be realized by choosing appropriate experimental parameters from^58,59^. The results show that the presence of OPA and strong Coulomb coupling enhances the quantum correlations between the two mechanical oscillators, and Coulomb interactions are more prominent in quantum correlations.

We next examine the effect of the phase of the optical field driving the OPA on quantum correlations between the two mechanical oscillators separated in space. In Fig. 4, we plot the quantum correlations, e.g., logarithmic negativity $E_N$, quantum steering ($S^{A\rightarrow B}: S^{B\rightarrow A}$), and quantum discord ($D_{A}:D_{B}$) as a function of the normalized detuning $\Delta /\omega _m$ for different values of a phase of the optical field driving the OPA (a) $\theta =\pi /16$, (b) $\theta =\pi /8$ , (c) $\theta =\pi /4$, and (d) $\theta =\pi /3$. As shown in Fig. 4a–d, it can be seen that the entanglement, quantum steering, and quantum discord larger with the phase  $\theta$ increases with fixed values of the nonlinear gain of the OPA $(G_a)$ and Coulomb coupling strength $\eta$. Because the proper choice of a phase of the optical field driving the OPA may lead to maximum noise suppression, thereby resulting in maximum entanglement, quantum steering, and quantum discord between the two mechanical oscillators. Besides, the optimum values of quantum steering and quantum discord are occurred at $\Delta /\omega _m=0.13$, and $\Delta /\omega _m=0.05$, respectively, for the case $\theta =\pi /3$. Furthermore, the quantum correlation between the two mechanical oscillators increases as the phase of the optical field driving the OPA ($\theta$) increases. Thus, we deduced that the presence of phase fluctuation of driving fields affects the quantum correlations.

It is also significant to study the robustness of quantum correlations between the two mechanical oscillators against temperature. Figure 5 shows plots of quantum correlations, e.g., logarithmic negativity ($E_N$), steering ($S^{A\rightarrow B}: S^{B\rightarrow A}$), and quantum discord ($D_{A}:D_{B}$) as a function of the temperature for different values drive power  (a) $P=50~\textrm{mW}$, (b) $P=70~\textrm{mW}$, (c) $P=80~\textrm{mW}$, and (d) $P=100~\textrm{mW}$ with $\Delta =0.6\omega _m$. It is obvious that quantum correlations, such as entanglement, quantum steering, and quantum discord, become stronger as the laser power increases. The reason for this is that increasing the laser power causes a stronger coupling between the mechanical oscillator$_{-}1$ and the cavity field due to an increase in the photon number in the cavity. The amount of entanglement, quantum steering, and quantum discord also decreases monotonically as the temperature increases because thermal noise in the environment induces decoherence. To be more specific, the entanglement degrades to zero, this is the phenomenon of entanglement of sudden death^60^. According to these results, an increase in temperature leads to the transition from quantum to classical regimes, which is caused by thermal fluctuations. As a result, the hybrid system does not exhibit any entanglement in classical regimes, despite the presence of quite strong laser power. We deduced that the stronger the thermal noise, the higher the temperature of the environment. The entanglement between two separated oscillators is then submerged by the strong thermal noise^50^. We note that the two-way steerable state vanishes with higher values of the environmental temperature. In addition, we obtain better quantum discord at low temperatures, and even at higher values of temperature, quantum discord is found to persist, although entanglement $E_N$ vanishes completely, indicating that quantum discord extends beyond entanglement and confirms the robustness of this measure against the fluctuations of the bath environment. Therefore, thermal fluctuations affect quantum correlation, while laser power enhances quantum correlation. We believe that these are appropriate measures to quantify quantum entanglement, quantum steering, and quantum discord and show that our proposed scheme enhances the quantum correlation and proves robust against fluctuations in the bath environment.

Furthermore, we can summarize Figs. 2, 3, 4 and 5 show that entanglement, quantum steering, and quantum discord all behave in the same way. As can be seen, quantum steering is bounded by quantum entanglement. We have $S^{A\rightarrow B}=S^{B\rightarrow A} > 0$ and logarithmic negativity $E_N > 0$ is the witness of two-way steering, while for $S^{A\rightarrow B}=S^{B\rightarrow A} =0$ and $E_N > 0$, the two mechanical oscillators are not steerable (i.e. no-way steering). Thus, the quantum discord is more robust than entanglement as shown in Fig. 2, 3, 4 and 5, because when $0 \le D_{A}< 1:0 \le D_{B} < 1$ (i.e. the two mechanical oscillators are separable (if $E_N = 0$) or entangled (if $E_N > 0$). On the other hand when $D_{A}> 1: D_{B}> 1$ (i.e. the two mechanical oscillators must be entangled) and we have $E_N > 0$. Moreover, we can also see from Figs. 2, 3, 4 and 5, that the quantum discord is more dominant than entanglement and also is a good quantifier of quantum correlation. The presence of OPA and strong Coulomb coupling enhances the quantum correlations between the two mechanical oscillators, and Coulomb interactions are more prominent in the nano-electro-optomechanical system. Such a phenomenon is because the enhancement of the effective coupling accelerates the quantum correlation in hybrid systems. Thus, entanglement detection is still a challenge experimentally, but quantum correlation detection is relatively easy. At present, some promising schemes have been suggested in^26,45^, so we can employ homodyne measurement techniques indirectly to detect quantum correlation especially the quantum entanglement^41,61^. Therefore, we believe that our scheme will be experimentally feasible with the quantum information experimental applications, and demand for the development of skills in quantum state manipulation.

## Conclusions

In conclusion, we have studied the quantum correlation in a nano-electro-optomechanical system enhanced by an optical parametric amplifier and Coulomb-type interaction. We consider a hybrid system that comprises a cavity and two charged mechanical oscillators with an OPA, in which the cavity mode is coupled to a charged mechanical oscillator via radiation pressure, and the two charged mechanical oscillators are coupled via Coulomb interaction. We showed that the Coulomb interaction between mechanical oscillators is the primary reason for the existence of a quantum correlation between the two mechanical oscillators. Our result shows that the presence of OPA and strong Coulomb coupling enhances the quantum correlations between the two mechanical oscillators. In addition, Coulomb interactions are more prominent in quantum correlations. Besides, in the presence of OPA, the maximum amount of quantum entanglement, quantum steering, and quantum discord achieved between the two mechanical oscillators than in the absence of OPA. This is because increasing the nonlinear gain of the OPA increases the photon number in the cavity, which leads to a stronger radiation pressure acting on the mechanical oscillator$_{-}1$. Additionally, we show that a proper phase choice of the optical field driving the OPA enhances quantum correlations under suitable conditions. This is because the proper choice of a phase of the optical field driving the OPA may lead to maximum noise suppression, thereby resulting in maximum quantum correlations. Furthermore, the quantum correlations decline rapidly with increasing temperature as a result of decoherence. Specifically, we noted that the quantum entanglement degrades to zero; this is the phenomenon of the quantum entanglement of sudden death. According to these results, an increase in temperature leads to the transition from quantum to classical regimes, which is caused by thermal fluctuations. Furthermore, when compared to quantum entanglement, the two-way steerable state vanishes with higher values of the environmental temperature. Finally, we have shown that quantum discord persists at higher temperature values, even though quantum entanglement disappears completely at higher temperature values. In this regard, quantum discord extends beyond entanglement and confirms the robustness of this measure against the fluctuations of the bath environment. Our proposed scheme of quantum correlation provides a promising platform for realizing continuous variable quantum information processing.

## Data Availability

The datasets used and/or analysed during the current study available from the corresponding author on reasonable request.
